# 126. Magnitude and Dynamics of the T-Cell Response to SARS-CoV-2 Infection and Vaccination

**DOI:** 10.1093/ofid/ofab466.126

**Published:** 2021-12-04

**Authors:** Thomas M Snyder, Rachel M Gittelman, Mark Klinger, Damon H May, Edward J Osborne, Ruth Taniguchi, H Jabran Zahid, Rebecca Elyanow, Sudeb C Dalai, Ian M Kaplan, Jennifer N Dines, Matthew T Noakes, Ravi Pandya, Lance Baldo, Simona Semprini, Claudio Cerchione, Fabio Nicolini, Massimiliano Mazza, Ottavia M Delmonte, Kerry Dobbs, Rocio Laguna-Goya, Gonazalo Carreño-Tarragona, Santiago Barrio, Luisa Imberti, Alessandra Sottini, Eugenia Quiros-Roldan, Camillo Rossi, Andrea Biondi, Laura Rachele Bettini, Mariella D’Angio, Paolo Bonfanti, Miranda F Tompkins, Camille Alba, Clifton Dalgard, Vittorio Sambri, Giovanni Martinelli, Jason D Goldman, James R Heath, Helen C Su, Luigi D Notarangelo, Estela Paz-Artal, Joaquin Martinez-Lopez, Jonathan M Carlson, Harlan S Robins

**Affiliations:** 1 Adaptive Biotechnologies, Seattle, Washington; 3 Microsoft Research, Redmond, Washington; 4 Adaptive Biotechnologies and Stanford University School of Medicine, Seattle, Washington; 5 Unit of Microbiology - The Great Romagna Hub Laboratory, Pievesestina ITALY and DIMES, University of Bologna, Bologna, Emilia-Romagna, Italy; 6 Istituto Scientifico Romagnolo per lo Studio e la Cura dei Tumori (IRST) IRCCS, Medola, Lombardia, Italy; 7 Immunotherapy, Cell Therapy and Biobank (ITCB), Istituto Scientifico Romagnolo per lo Studio e la Cura dei Tumori (IRST) IRCCS, Medola, Lombardia, Italy; 8 Immune Deficiency Genetics Section, Laboratory of Clinical Immunology and Microbiology, National Institute of Allergy and Infectious Diseases, National Institutes of Health, Bethesda, Maryland; 9 Department of Immunology, Hospital ^12^de Octubre, i+^12^, Madrid, Madrid, Spain; 10 Hematology Department, Hospital ^12^de Octubre, i+^12^, CNIO, Complutense University, Madrid, Madrid, Spain; 11 Laboratorio CREA, Department of Infectious and Tropical Diseases, and Medical Officer, ASST Spedali Civili di Brescia and University of Brescia, Brescia, Lombardia, Italy; 12 Department of Pediatrics and Centro Tettamanti-European Reference Network PaedCan, EuroBloodNet, MetabERN-University of Milano-Bicocca-Fondazione MBBM-Ospedale San Gerardo, Monza, Lombardia, Italy; 14 Department of Infectious Diseases, University of Milano-Bicocca-Ospedale San Gerardo, Monza, Lombardia, Italy; 15 The American Genome Center, Uniformed Services University of the Health Sciences, Bethesda, Maryland; 16 Swedish Medical Center, Seattle, WA, USA, and Division of Allergy and Infectious Diseases, University of Washington, Seattle, Washington; 17 Institute for Systems Biology, Seattle, WA, USA, Seattle, Washington; 18 National Institute of Allergy and Infectious Diseases, National Institutes of Health, Bethesda, MD

## Abstract

**Background:**

T cells are central to the early identification and clearance of viral infections and support antibody generation by B cells, making them desirable for assessing the immune response to SARS-CoV-2 infection and vaccines. We combined 2 high-throughput immune profiling methods to create a quantitative picture of the SARS-CoV-2 T-cell response that is highly sensitive, durable, diagnostic, and discriminatory between natural infection and vaccination.

**Methods:**

We deeply characterized 116 convalescent COVID-19 subjects by experimentally mapping CD8 and CD4 T-cell responses via antigen stimulation to 545 Human Leukocyte Antigen (HLA) class I and 284 class II viral peptides. We also performed T-cell receptor (TCR) repertoire sequencing on 1815 samples from 1521 PCR-confirmed SARS-CoV-2 cases and 3500 controls to identify shared public TCRs from SARS-CoV-2-associated CD8 and CD4 T cells. Combining these approaches with additional samples from vaccinated individuals, we characterized the response to natural infection as well as vaccination by separating responses to spike protein from other viral targets.

**Results:**

We find that T-cell responses are often driven by a few immunodominant, HLA-restricted epitopes. As expected, the SARS-CoV-2 T-cell response peaks about 1-2 weeks after infection and is detectable at least several months after recovery. Applying these data, we trained a classifier to diagnose past SARS-CoV-2 infection based solely on TCR sequencing from blood samples and observed, at 99.8% specificity, high sensitivity soon after diagnosis (Day 3–7 = 85.1%; Day 8–14 = 94.8%) that persists after recovery (Day 29+/convalescent = 95.4%). Finally, by evaluating TCRs binding epitopes targeting all non-spike SARS-CoV-2 proteins, we were able to separate natural infection from vaccination with > 99% specificity.

**Conclusion:**

TCR repertoire sequencing from whole blood reliably measures the adaptive immune response to SARS-CoV-2 soon after viral antigenic exposure (before antibodies are typically detectable) as well as at later time points, and distinguishes post-infection vs. vaccine immune responses with high specificity. This approach to characterizing the cellular immune response has applications in clinical diagnostics as well as vaccine development and monitoring.

**Disclosures:**

**Thomas M. Snyder, PhD**, **Adaptive Biotechnologies** (Employee, Shareholder) **Rachel M. Gittelman, PhD**, **Adaptive Biotechnologies** (Employee, Shareholder) **Mark Klinger, PhD**, **Adaptive Biotechnologies** (Employee, Shareholder) **Damon H. May, PhD**, **Adaptive Biotechnologies** (Employee, Shareholder) **Edward J. Osborne, PhD**, **Adaptive Biotechnologies** (Employee, Shareholder) **Ruth Taniguchi, PhD**, **Adaptive Biotechnologies** (Employee, Shareholder) **H. Jabran Zahid, PhD**, **Microsoft Research** (Employee, Shareholder) **Rebecca Elyanow, PhD**, **Adaptive Biotechnologies** (Employee, Shareholder) **Sudeb C. Dalai, MD, PhD**, **Adaptive Biotechnologies** (Employee, Shareholder) **Ian M. Kaplan, PhD**, **Adaptive Biotechnologies** (Employee, Shareholder) **Jennifer N. Dines, MD**, **Adaptive Biotechnologies** (Employee, Shareholder) **Matthew T. Noakes, PhD**, **Adaptive Biotechnologies** (Employee, Shareholder) **Ravi Pandya, PhD**, **Microsoft Research** (Employee, Shareholder) **Lance Baldo, MD**, **Adaptive Biotechnologies** (Employee, Shareholder, Leadership Interest) **James R. Heath, PhD**, **Merck** (Research Grant or Support, Funding (from BARDA) for the ISB INCOV project, but had no role in planning the research or in writing the paper.) **Joaquin Martinez-Lopez, MD, PhD**, **Adaptive Biotechnologies** (Consultant) **Jonathan M. Carlson, PhD**, **Microsoft Research** (Employee, Shareholder) **Harlan S. Robins, PhD**, **Adaptive Biotechnologies** (Board Member, Employee, Shareholder)

